# Persistance de la veine cave supérieure gauche: à propos d'un cas

**DOI:** 10.11604/pamj.2015.22.36.7861

**Published:** 2015-09-16

**Authors:** Kamel Abidi, Manel Jellouli, Yousra Hammi, Tahar Gargah

**Affiliations:** 1 Service de Pédiatrie, Hôpital Charles Nicolle de Tunis, Tunis, Tunisie

**Keywords:** Echographie, veine cave, malformation vasculaire, Ultrasound, vena cava, vascular malformation

## Abstract

La persistance de la veine cave supérieure gauche (PVCSG) est une malformation congénitale rare et bénigne. Elle est souvent asymptomatique et sa découverte est dans la majorité des cas fortuite. Nous rapportons le cas d'un enfant chez lequel on découvre cette anomalie suite à une perte de connaissance. S.M, âgé de 9 ans, sans antécédents pathologiques notables, admis pour perte de connaissance de durée brève, sans mouvements anormaux toniques ou cloniques. L'examen physique à son admission est normal. L’électrocardiogramme est sans anomalies. La radiographie du thorax a montré un arc moyen gauche en double contour. Le Holter rythmique a montré des signes d'hyperréactivité vagale. L’échocardiographie trans-thoracique (ETT) a mis en évidence une dilatation nette du sinus coronaire et a éliminé la présence d'une cardiopathie. Une angio- IRM cardiaque a confirmé le diagnostic de PVCSG. Par ailleurs l'aorte thoracique a été normale dans ces différents segments.

## Introduction

La persistance de la veine cave supérieure gauche (PVCSG) est une malformation congénitale rare et bénigne. Elle est souvent asymptomatique et sa découverte est dans la majorité des cas fortuite [1]. Nous rapportons ici le cas d'un enfant chez qui on a découvert cette anomalie suite à une perte de connaissance.

## Patient et observation

**Figure 1:**
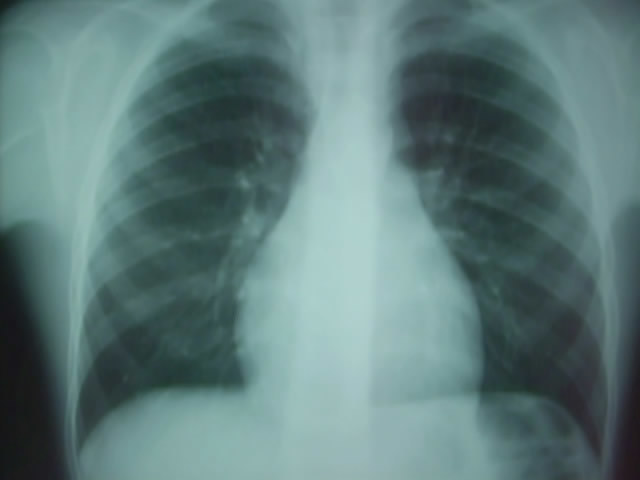
Radiographie du thorax montrant un arc moyen gauche en double contours

**Figure 2:**
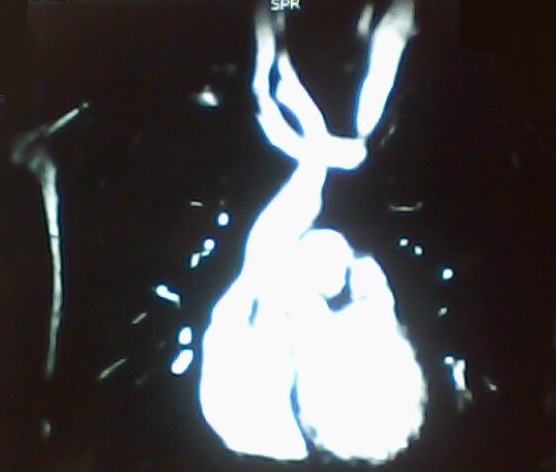
IRM cardiaque en reconstruction coronale montrant la veine cave supérieure gauche avec abouchement dans le sinus coronaire

**Figure 3:**
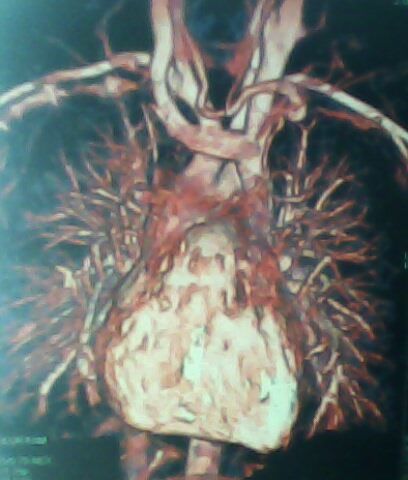
IRM cardiaque en reconstruction volumique montrant la veine cave supérieure gauche avec absence de la veine cave supérieure droite

S.M, âgé de 9 ans, sans antécédents pathologiques notables, admis pour perte de connaissance de durée brève, sans mouvements anormaux toniques ou cloniques. L'examen physique trouve un enfant eutrophique pour l’âge. Il ne présente pas de déformation thoracique, ni de cyanose, ni d'hippocratisme digital. Il n'a pas de signes d'insuffisance cardiaque. L'examen cardio-vasculaire est normal: il n'y a pas de souffle, ni de bruit surajouté. L'examen neurologique est normal. Le reste de l'examen est sans anomalie. L’électrocardiogramme objectivait un rythme cardiaque sinusal avec absence de troubles de conduction et de repolarisation. Le Holter rythmique a montré des signes d'hyperréactivité vagale marquée par une instabilité du rythme et des ralentissements brutaux de la fréquence cardiaque. La radiographie du thorax a montré un arc moyen gauche en double contour (Figure 1). L’échocardiographie trans-thoracique (ETT) a mis en évidence une dilatation nette du sinus coronaire et a éliminé la présence d'une cardiopathie. Une angio-IRM cardiaque (Figure 2, Figure 3) a confirmé la présence de la veine cave supérieure gauche communiquant avec un sinus coronaire dilaté qui se diverse dans l'oreillette droite. La veine cave supérieure droite n’était pas décelable. Il n'y a pas de signes en faveur d'un retour veineux pulmonaire anormal et les artères pulmonaires droites et gauches sont de calibre et de trajets normaux, convergeant vers une oreillette gauche de morphologie et de taille normales. Par ailleurs l'aorte thoracique a été normale dans ces différents segments.

## Discussion

La PVCSG est une anomalie rare de l'organogenèse due à la persistance de la partie terminale de la veine cardinale antérieure gauche, dont l'involution intervient normalement au sixième mois de la vie utérine [2]. Souvent, les deux veines caves supérieurs sont présentes et communiquent souvent par des anastomoses médiastinales ou par un tronc veineux innominé. L'absence de la veine cave droite est extrêmement rare [3, 4], comme ce fut le cas chez notre patient, et la veine cave supérieure gauche draine tout le sang veineux de la tête et des membres supérieurs. Cette anomalie peut être isolée ou le plus souvent associée à une cardiopathie congénitale. Dans notre cas, les explorations n'ont décelées aucune anomalie en dehors de la PVCSG. En dehors d'une cardiopathie congénitale plus ou moins complexe, les patients porteurs de cette malformation sont souvent asymptomatiques [5]. Le diagnostic se fait habituellement devant une dilatation du sinus coronaire à l’échographie cardiaque trans-thoracique ou lors de la pratique d'un cathétérisme veineux central [6, 7]. Dans certains cas, cette anomalie peut être à l'origine d'une cyanose lorsque le retour se fait au niveau de l'oreillette gauche avec le risque d'embolie paradoxale [6]. Dans notre cas, l'enfant présentait une perte de connaissance qui pourrait être expliqué par la dilatation du sinus coronaire peut être à l'origine d'hypertonie vagale avec des troubles de rythme. Le diagnostic se fait généralement de façon fortuite.

## Conclusion

La PVCSG est une anomalie rare du retour veineux systémique. Son diagnostic doit être suspecté en cas de visualisation d'un sinus coronaire dilaté par ETT et doit être confirmé par l'angio-IRM cardiaque.
